# On Modern Progress in Ophthalmic Medicine and Surgery

**Published:** 1894-07-21

**Authors:** Robert Brudenell Carter

**Affiliations:** Consulting Ophthalmic Surgeon to St. George's Hospital


### On Modern Progress in Ophthalmic Medicine and Surgery.

By Robert Brudenell Carter, F.R.C.S., Consulting Ophthalmic Surgeon to St. George's Hospital.

### LAMINAR CATARACT.

Besides the form of cataract incidental to old age, there is yet another which is of somewhat frequent occurrence, and which depends upon arrested or imperfect development of the lens at some period prior to birth. The result is that a single lamina, or layer, of the lens, is of imperfect transparency; and the precise diameter of this cloudy lamina, as well as the degree or depth of its cloudiness, will differ considerably in different people. "Laminar" cataract, as it is called, may be almost equal in diameter to the lens itself, or may be limited to a small central area. It may be of such density as to occasion a defect of vision almost amounting to blindness, or it may be so slight a cloud as to escape the observation of parents or teachers of only average carelessness. The child with laminar cataract sees badly at a distance, and brings small objects close to his eyes in order to obtain larger retinal images, so that he is often supposed to be "short-sighted." If the cloudy area be small, he will see fairly well through the clear margins of his lenses when his pupils are moderately dilated; and therefore, like many of the subjects of senile cataract, he will see better when his face is turned away from the light. Glasses are seldom helpful to him, and when he is examined with the ophthalmoscope, or by focal illumination, with moderately dilated pupils, the cloudy laminæ will be clearly seen. When very small and central, the cloud may be of irregular outline; but when of moderate size, it is always circular, with or without processes of opacity radiating from it, like the spokes of a wheel. As a general rule, to which there are exceptions, the opacity will be of uniform depth over its whole extent.

The defect of vision produced by laminar cataract is seldom noticed until the subject of it is eight years old or more, and the treatment formerly in vogue was to keep the pupils dilated by some preparation of belladonna, so that the subject should be enabled to see through the clear margins of his lenses around the opacity. Even prior to the time when the effect of a dilated pupil in producing increased tension became generally known, this method was seen to be attended with numerous disadvantages. The preparations of belladonna are active poisons, the regular use of which by young people could hardly fail to be productive of occasional accidents; while there would be many places and positions in which they would not readily be procurable in a state of purity. Many eyes become intolerant of them after a time, so that they have to be laid aside on account of swelling and surface irritation. When the operation of iridectomy came into use for other purposes, it was soon suggested that by its means the margin of the lens might be permanently uncovered over a sufficient area; but it was found in practice that the optical results of this profeeding were often disappointing. In ordinary iridectomy, the piece of iris removed is much broader at the periphery than at the margin of the pupil; and the extreme edge of the lens, which is thus uncovered over a considerable part of its circumference, is often of irregular curvature, and is productive of an amount of spherical aberration which interferes with vision. The late Mr. Critchett set himself to overcome this difficulty, and devised an operation of great ingenuity, to which he gave the name of iriddesis. He made an incision into the anterior chamber, just outside the margin of the true cornea, with a narrow two-edged knife or cutting needle, which was carried into the chamber by a single thrust and at once withdrawn, little or no escape of aqueous humour being permitted. A tiny loop of the finest black suture silk, with its ends twisted into the first stage of a knot, was then laid upon the surface of the eyeball so as to surround the wound. A Tyrrell's hook, or a fine pair of canula forceps, was next carried through the loop and the wound into the anterior chamber, and made to seize and withdraw the iris to the desired extent. When this was done, an assistant, armed with two pairs of forceps, picked up the two ends of the silk, and tightened the loop around the prolapsed iris. If only a very small displacement were desired, canula forceps would be used, the iris seized about midway between the periphery and the pupillary margin, and the whole of the latter left within the eye; while, for a more considerable effect, the Tyrrell's hook would be passed over the margin of the pupil, and the complete width of the iris would be withdrawn. Whichever was done, the opposite side of the iris was pulled somewhat over the central opacity towards the seat of operation, and the clear portion of the lens was left exposed by a triangular opening, which had its base towards the natural pupil, and its apex towards the circumference of the anterior chamber. The immediate visual results were excellent, and the beauty and delicacy of the operation commanded the admiration of all those who saw it. and still more of all those who were sufficiently skilful to practise it. It was attended by the obvious disadvantage of requiring the co-operation of two surgeons of at least equal dexterity, the tightening of the ligature requiring at least as much skill as the seizure and withdrawal of the iris. This requirement was easily supplied in London, but presented greater difficulties in the provinces; and, being at that time surgeon to a country eye hospital, I found it necessary to contrive an instrument by means of which I could apply and tighten the ligature with one hand, while the other was engaged in the manipulation and control of the iris. Before long, however, unexpected objections to the operation began to force themselves into notice. It was not thoroughly understood at that period that adhesions which check the free movements of the iris are always sources of danger from their liability to excite recurrent inflammation; but it was found, after a while, that a large proportion of the patients who had been operated upon by Mr. Critchett's method, after enjoying excellent vision for a longer or shorter time, became the subjects of severe forms of iritis, which proved exceedingly rebellious to treatment, and, in a corresponding degree, liable to return after apparent recovery. Disasters of this kind became so frequent that it was impossible to overlook the dangers which iriddesis entailed, and, after having been much employed during two or three years, the operation rapidly fell into complete disuse. Mechanically it was a masterpiece of ingenuity and an absolute success; but surgically it was a pronounced failure. I am acquainted with a gentleman upon whom the late Mr. Critchett performed it five and twenty years ago, in whom no trouble has arisen, and who sees well by its means; but I have neither done it myself nor seen it done by others, for almost the same period of time.

